# Detection of biomarkers for Hepatocellular Carcinoma using a hybrid univariate gene selection methods

**DOI:** 10.1186/1742-4682-9-34

**Published:** 2012-08-06

**Authors:** Nagwan M Abdel Samee, Nahed H Solouma, Yasser M Kadah

**Affiliations:** 1 Computer Engineering Department, Misr University for Science and Technology, Giza, Egypt; 2 Engineering Applications Department, NILES, Cairo University, Giza, Egypt; 3 Biomedical Engineering Department, Cairo University, Giza, Egypt

## Abstract

**Background:**

Discovering new biomarkers has a great role in improving early diagnosis of Hepatocellular carcinoma (HCC). The experimental determination of biomarkers needs a lot of time and money. This motivates this work to use *in-silico* prediction of biomarkers to reduce the number of experiments required for detecting new ones. This is achieved by extracting the most representative genes in microarrays of HCC.

**Results:**

In this work, we provide a method for extracting the differential expressed genes, up regulated ones, that can be considered candidate biomarkers in high throughput microarrays of HCC. We examine the power of several gene selection methods (such as Pearson’s correlation coefficient, Cosine coefficient, Euclidean distance, Mutual information and Entropy with different estimators) in selecting informative genes. A biological interpretation of the highly ranked genes is done using KEGG (Kyoto Encyclopedia of Genes and Genomes) pathways, ENTREZ and DAVID (Database for Annotation, Visualization, and Integrated Discovery) databases. The top ten genes selected using Pearson’s correlation coefficient and Cosine coefficient contained six genes that have been implicated in cancer (often multiple cancers) genesis in previous studies. A fewer number of genes were obtained by the other methods (4 genes using Mutual information, 3genes using Euclidean distance and only one gene using Entropy). A better result was obtained by the utilization of a hybrid approach based on intersecting the highly ranked genes in the output of all investigated methods. This hybrid combination yielded seven genes (2 genes for HCC and 5 genes in different types of cancer) in the top ten genes of the list of intersected genes.

**Conclusions:**

To strengthen the effectiveness of the univariate selection methods, we propose a hybrid approach by intersecting several of these methods in a cascaded manner. This approach surpasses all of univariate selection methods when used individually according to biological interpretation and the examination of gene expression signal profiles.

## Background

Microarray is a powerful technology for gene profiling. Being able to retrieve the gene expression values of the whole genome and thus studying the molecular biology of tissues can lead to diagnosis of different diseases [1]. Next to the value of microarrays in understanding the biological processes underlying a specific disease, it has a great role in discovering new biomarkers for cancers. A biomarker in the biomedical field is a substance that has a characteristic feature as an objective indicator of a biological state, such as normal physiological processes [2]. The most important advantage of Insilco prediction of biomarkers using microarrays is minimizing the number of experimental work required for detecting new biomarkers and thus saving much time and money.

In liver cancer, exploring gene expression patterns of samples from healthy patients and others infected with Hepatocellular Carcinoma (HCC) has revealed a significant difference in the expression of some genes from normal to tumour samples. Genes having high variance between both classes of samples in their expression are informative features that should be used in any further analysis as suggested biomarkers. Discovering new biomarkers of HCC can help in early detection of this type of cancer. Early detection of HCC is a vital issue as it can help patients in receiving therapeutic benefits rather than curative surgery. Unfortunately, there is no effective biomarker for early detection of HCC. The current diagnosing of HCC relies on detection of an inaccurate biomarker, alpha-fetoprotein (AFP) [3]. Therefore, there is a need for detecting new reliable biomarkers for HCC.

Detection of biomarkers in the context of machine learning can be treated as a feature selection problem that tries to select features (markers) that can distinguish between different classes of data. The selected features are a list of genes that might be informative for discriminating different types and subtypes of diseases. So we will refer to feature selection all through this paper by gene selection.

A widely used approach for gene selection is the univariate selection approach [4,5]. Univariate selection approaches yield consistently better results than multivariate approaches [6]. In the univariate approach, the relevance of each gene is determined individually. However, the multivariate approach such as Singular Value Decomposition (SVD) and Principal Component Analysis (PCA) considers the interactions between genes. Some of the univariate methods are: Pearson’s correlation coefficients, Euclidean distance, Cosine coefficient, Entropy and Mutual information.

Discovery of biomarkers from microarrays data was studied in several publications [4-7]. All of these research efforts were seeking for selecting informative genes as a prerequisite step of a high performance classifier. None have assessed the quality of the gene selection methods from the point of retrieving relevant biomarkers known in the biological databases. A research paper made a comparison between the univariate and multivariate approaches in improving the accuracy of classification [6]. This work employed seven microarrays datasets representing different types of cancer. They found that the univariate methods surpassed the multivariate with five datasets. This was due to the small number of samples relative to the number of genes being studied which is always the case in microarrays. Another research work offered a combination between the Correlation Coefficient, as a univariate selection approach, and the singular value decomposition (SVD), as a multivariate approach [4]. However, they did not attain high classification accuracy due to the dependency between samples of the microarrays data being studied. So in our research, we focused on testing the Correlation Coefficient as a gene selection method individually or integrated with other univariate gene selection methods. A robust method of discovering new biomarker using the ensemble feature selection techniques in support vector machine (SVM) classifier was also presented [2]. This method improved the performance of the classification of microarrays data. However this was achieved only in cases when studying a few tens of genes. So, this method is not suitable for studying high throughput microarrays data that almost contain more than 20,000 genes. Another study by Cho et al. [5] presented a comparative study of different gene selection methods (Pearson’s correlation coefficients, Euclidean distance, Cosine coefficient, Mutual information and information gain). They used three benchmarking datasets and tested the impact of using such gene selection methods on different classifiers. An ideal biomarker was assumed that had two values of 0 and 1, which might produce irrelevant correlation and cosine coefficient. So, in this study we rather preferred to propose different values than those ones as well as normalize the gene expression values when the Euclidean Distance and the Cosine Coefficient are being calculated.

To conclude, the scope of those previous publications was focused on benchmarking data sets, which contains a few thousands of genes, to compare the adequacy of different gene selection methods and usually assessed using different classifiers. Therefore, we were motivated to expand the research to cover microarrays of HCC comprising a huge number of genes by means of the proposed hybrid technique. We examined the power of some of the previous methods (cf. [5]) in addition to different Entropy estimators. Biological validation of the retained top ten lists of genes is proposed in our work too. Moreover, the significance of signal profiles of the selected genes in both normal and tumor samples were validated using *t*-test.

Briefly, we defined an ideal biomarker as a gene that has two discrete values, minus one in normal samples and one in tumour samples. Genes with similar profile to the ideal biomarker are selected using Pearson’s correlation coefficients, Euclidean distance, Cosine coefficient, Entropy and Mutual information. Then they are ranked according to their similarity with the ideal gene. Highly ranked genes from all methods are intersected in a cascaded manner. Finally, the top ranked genes are examined by checking their signal profiles and mining the biological databases for their existence as known biomarkers.

## Methods

**Figure 1:**
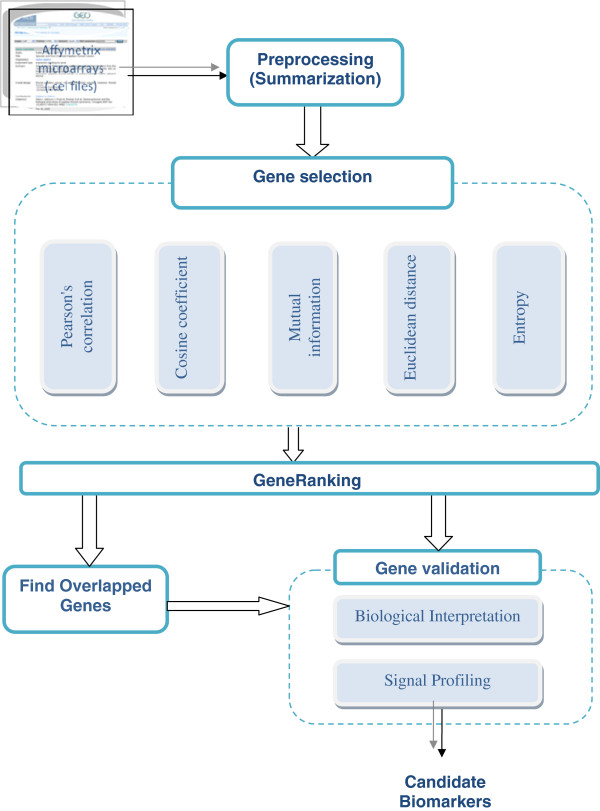
The proposed hybrid technique.

The steps of the proposed framework include pre-processing of Affymetrix files, gene selection, gene ranking, finding common genes and gene validation through biological interpretation and signal profiling. These steps are shown in Figure 1.

### Materials

Thirty five microarray samples were downloaded from Gene Expression Omnibus (GEO) [8]. Nineteen of theses samples are taken from normal subjects. The remaining sixteen samples are for subjects with HCC as a complication of HCV cirrhosis. This data were collected on the Affymetrix HG-U133A 2.0 platform. The raw data in “.CEL” format was collected from GEO are pre-processed using the Affy package provided by Bioconductor [9].

### Ideal biomarker

**Figure 2:**
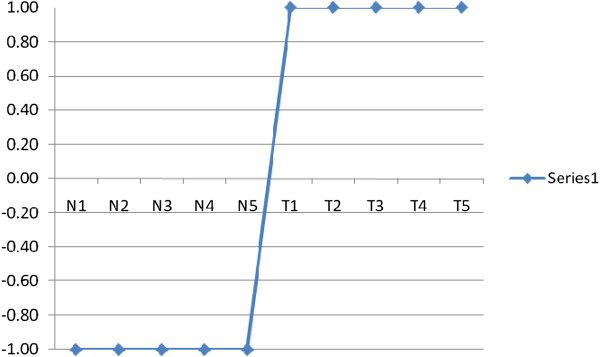
An ideal up regulated biomarker.

Suppose we have a gene expression matrix that contains a set of genes' expression values measured in *Q* samples. The first *k* samples are taken from normal persons and the remaining *L* samples are taken from HCC patients. Each gene in this matrix can be defined as a vector of *Q* values. An ideal up regulated biomarker can be defined as a gene that having a value of minus one in normal samples and a value of one in tumour samples. The ideal up regulated biomarker is a step up signal as shown in Figure 2.

### Pearson's correlation coefficient

The similarity between the ideal up regulated biomarker, *B*_*Ideal*_, and a gene Y can be determined by computing the Pearson's correlation coefficient [10] as stated in Equation 1.

(1) $$r=\frac{\sum_{i=1}^{Q}{(Y}_{i}-\bar{Y}{)(\text{B}}_{{\text{Ideal}}_{\text{i}}}-\bar{B_{\text{Ideal}}})}{\sqrt{\sum_{i=1}^{Q}{{(\text{Y}}_{i}-\bar{Y})}^{2}}\sqrt{\sum_{i=1}^{Q}{{(B}_{{\text{Ideal}}_{i}}-\bar{B_{\text{Ideal}}})}^{2}}}$$

The Pearson's correlation coefficient, r, is always between −1 and +1. The closer the correlation is to +/−1, the closer to a perfect linear relationship.

### Euclidean distance

The similarity between two vectors can be determined by measuring the distance between them in the space. So, we used the Euclidean distance between *B*_*Ideal*_ and all genes as another measure of similarity between them. See the following equation.

(2) $$d=\sum_{i=1}^{Q}{{((Y}_{i}-\overset{-}{Y}{)\text{ -B}}_{{\text{Ideal}}_{i}})}^{2}$$

The gene expression values were first normalized (by subtracting the mean value of each gene from all its values) before calculating the Euclidean distance between them and *B*_*Ideal*_. In this manner, all values will be in the same range from −1 to 1 and an accurate distance will be calculated.

### Cosine coefficient

The Cosine coefficient can measure the dependency between *B*_*Ideal*_, and a gene Y as seen in Equation 3. If the Cosine coefficient is zero, then they are independent and if one, then they are pointing in the same direction.

(3) $$r_{\text{cosine}}=\frac{\sum_{i=1}^{Q}{(Y}_{i}-\bar{Y}{)\text{ B}}_{{\text{Ideal}}_{i}}}{\sqrt{\sum_{i=1}^{Q}{{(Y}_{i}-\bar{Y})}^{2}}\sqrt{\sum_{i=1}^{Q}B_{{\text{Ideal}}_{i}}{}^{2}}}$$

In the same manner, all genes expression values were first normalized as mentioned above.

### Mutual information

Informative genes can also be discovered by computing the Mutual information between all genes and *B*_*Ideal*_. The formula of calculating the Mutual information [11,12] is given as,

(4) $${\text{I(B}}_{\text{Ideal}}\text{,Y})=H(B_{\text{Ideal}})-H(B_{\text{Ideal}}|Y)$$

In this formula, we need firstly need to calculate $H(B_{\text{Ideal}})$, the entropy of *B*_*Ideal*_, and the conditional entropy between *B*_*Ideal*_ and a gene Y: $H(B_{\text{Ideal}}|Y)$.

### Entropy

The Entropy is a measure of the uncertainty of a discrete random variable x. It is calculated via estimators. The estimator that is most commonly used is the Empirical estimator. It is also called Maximum likelihood [13]. It is defined by,

(5) $$H^{\text{emp}}(X)=-\sum_{k=1}^{n}\frac{nb(x_{k})}{Q}\log\frac{nb(x_{k})}{Q}$$

where, $nb(x_{k})$ is the number of data points in bin k, m is number of observations (the number of gene expression values) and n is the number of bins. This method estimates the probability of each gene by counting the number of values in each bin of comparable values. In this paper, the number of bins equals two as the informative genes should have different expression values in normal samples than those in tumour samples. Other entropy's estimators are: Miller-Madow, Shrink and Schurmann-Grassberger. The Miller-Madow Entropy estimator adds a correction bias to the Empirical estimator. The Shrinkage Entropy estimator is a combination of two different estimators: one with low variance and the other with low bias. The last examined estimator is the Schurmann-Grassberger Entropy estimator. It uses the Dirichlet distribution which is a generalization of Beta distribution [13-16].

## Results

For each gene selection method, we calculated the similarity between the ideal gene and 22277 genes comprising the original data set. Genes that have a strong association with the ideal gene should have Pearson correlation coefficient values from ±0.7-1 [5]. So, the number of retrieved genes using this method was 2284 genes. We restricted the number of selected genes using the other methods to the same number of genes. The functional annotations of the top ten genes are mined through DAVID's functional annotation [17], ENTREZ [18] and KEGG pathways [19]. The results obtained from each method are listed in Tables 1, 2, 3, 4 and 5. In each table, we listed for each gene in the top ten lists its Affy ID, symbol, ENTREZ ID, type, Gene Ontology (GO) processes, Oncology term and pathways. Signal profiles of first four genes in the top ten genes are charted as shown in the figures section.

**Table 1:** Functional annotation of the top ten genes selected using Pearson's correlation coefficient and Cosine coefficient

**Affy ID**	**Gene symbol**	**Entrez gene ID**	**Gene type**	**GO processes**	**Oncology**	**KEGG pathways**
202988_s_at	RGS1	5996	Protein coding	Immune response		CXCR4-mediated signaling events, organism-specific biosystem Pathway Interaction Database ID: [cxcr4_pathway]
208949_s_at	LGALS3	3958	Protein coding	Immune response, cell adhesion, T-cell regulation	Skin cancer, Breast cancer, Colon cancer, Lung cancer, Cervical cancer, Colorectal cancer, Bladder cancer, Thyroid cancer, Pancreatic cancer, prostate cancer, HCC	Immune System, organism-specific biosystem REACTOME ID: [REACT_6900]
217028_at, 211919_s_at	CXCR4	7852	Protein coding	inflammatory response, response to viral	prostate cancer, kidney cancer, Breast cancer, Lung cancer, bladder cancer, Pancreatic cancer, Colorectal cancer, Thyroide cancer, Qastric cancer	CXCR4-mediated signaling events, organism-specific biosystem Pathway Interaction Database ID: [cxcr4_pathway]
205798_at	IL7R	3575	Protein coding	Immune response	HCV	Primary immunodeficiency KEGG ID: [hsa05340]
202157_s_at	CELF2	10659	Protein coding	Regulation of heart contraction	Breast cancer, Colon Cancer	mRNA processing, organism-specific biosystemWikiPathways ID: [WP411]
209606_at	CYTIP		Protein coding	Regulation of cell adhesion		
202992_at	C7	730	Protein coding	Immune response	Hepatoma cell lined	Immune System, organism-specific biosystem REACTOME ID: [REACT_6900]
211813_x_at, 201893_x_at	DCN	1634	Protein coding	Cell growth	Colon Carcinoma, Breast Cancer	(TGF-beta signaling pathway) KEGG ID: [hsa04350]

**Table 2:** Functional annotation of the top ten genes selected using Mutual information

**Affy ID**	**Gene symbol**	**Entrez gene ID**	**Gene type**	**GO processes**	**Oncology**	**KEGG pathways**
201034_at	ADD3	120	Protein coding	Drug response		
201141_at	GPNMB	10457	Protein coding	negative regulation of cell proliferation	Liver inflammation	
201278_at, 201280_s_at	DAB2	1601	Protein coding		Prostate Cancer, Breast Cancer, esophageal Cancer	
201311_s_at	SH3BGRL	6451	Protein coding			EGFR1 Signaling Pathway, organism-specific biosystemWikiPathways ID: [WP437]
201893_x_at	DCN	1634	Protein coding	Cell growth	Colon Carcinoma, Breast Cancer	(TGF-beta signaling pathway) KEGG ID: [hsa04350]
202157_s_at	CELF2	10659			Breast cancer, Colon Cancer	
202207_at	Arl4c	10123	Protein coding			
202336_s_at	Pam	5066	Protein coding	Response to drug		
202403_s_at	COL1A2	1278	Protein coding	blood vessel development	colorectal cancer, breast cancer	Platelet Activation, organism-specific biosystem REACTOME ID: [REACT_798]

**Table 3:** Functional annotation of the top ten genes selected using Euclidean distance

**Affy ID**	**Gene symbol**	**Entrez gene ID**	**Gene type**	**GO processes**	**Oncology**	**KEGG pathways**
201893_x_at, 211896_s_at, 211813_x_at	DCN	1634	Protein coding	Cell growth	Colon Carcinoma, Breast Cancer	(TGF-beta signaling pathway) KEGG ID: [hsa04350]
202992_at	C7	730	Protein coding	Immune response	Hepatoma cell lined	Immune System, organism-specific biosystem REACTOME ID: [REACT_6900]
201918_at	SLC25A36,	55186,	Protein coding			
204319_s_at	Rgs10,	6001	Protein coding			G alpha (i) signalling events, organism-specific biosystem REACTOME ID: [REACT_19231]
211368_s_at	CASP1	834	Protein coding	plays a central role in the execution-phase of cell apoptosis.	Colorectal cancer	Immune System, organism-specific biosystem REACTOME ID: [REACT_6900]
218589_at	LPAR6	10161		The protein encoded by this gene belongs to the family of G-protein coupled receptors, that are preferentially activated by adenosine and uridine nucleotides.		G alpha (q) signalling events, organism-specific biosystem REACTOME ID: [REACT_18283]
212509_s_at	mxra7	439921	Protein coding			
201278_at	DAB2	1601	Protein coding	The down-regulation of DAB2 may play an important role in ovarian carcinogenesis.	Prostate cancer, breast cancer	Endocytosis, organism-specific biosystem KEGG ID: [hsa04144]

**Table 4:** Functional annotation of the top ten genes selected using Empirical estimator of Entropy

**Affy ID**	**Gene symbol**	**Entrez gene ID**	**Gene type**	**GO processes**	**Oncology**	**KEGG pathways**
117_at	HSPA7	3310	Protein coding	Response to stress		MAPK signalling pathway KEGG ID: [hsa04010]
1438_at	EPHB3	2049	Protein coding	central nervous system projection neuron axonogenesis		
200033_at	DDX5	1655	Protein coding	Cell growth	prostate cancer	Direct p53 effectors, organism-specific biosystem Pathway Interaction Database ID: [p53downstreampathway]
200055_at	taf10	6881	Protein coding	Viral reproduction		HIV Infection, organism-specific biosystem REACTOME ID:
200060_s_at	LOC643446	10921	Protein coding	Metabolic process		Metabolism of RNA, organism-specific biosystem REACTOME ID: [REACT_21257]
200068_s_at	Canx	821	Protein coding			Immune System, organism-specific biosystem REACTOME ID: [REACT_6900]
200087_s_at	Tmed2	10959				
200593_s_at	Hnrnpu,	3192				
200606_at	Dsp	1832		Wound healing		
200610_s_at	Ncl	4691				

**Table 5:** Functional annotation of the top ten genes selected using the hybrid technique

**Affy ID**	**Gene symbol**	**Entrez gene ID**	**Gene type**	**GO processes**	**Oncology**	**KEGG pathways**
208949_s_at	LGALS3	3958	Protein coding	Immune response, cell adhesion, T-cell regulation	Skin cancer, Breast cancer, Colon cancer, Lung cancer, Cervical cancer, Colorectal cancer, Bladder cancer, Thyroid cancer, Pancreatic cancer, prostate cancer, HCC	Immune System, organism-specific biosystem REACTOME ID: [REACT_6900]
202157_s_at	CELF2	10659	Protein coding	Regulation of heart contraction	Breast cancer, Colon Cancer	mRNA processing, organism-specific biosystem WikiPathways ID: [WP411]
209606_at	CYTIP		Protein coding	Regulation of cell adhesion		
202992_at	C7	730	Protein coding	Immune response	Hepatoma cell lined	Immune System, organism-specific biosystem REACTOME ID: [REACT_6900]
211813_x_at, 211896_s_at, 201893_x_at	DCN	1634	Protein coding	Cell growth	Colon Carcinoma, Breast Cancer	(TGF-beta signaling pathway) KEGG ID: [hsa04350]
211919_s_at	CXCR4	7852	Protein coding	inflammatory response, response to viral	HCC, prostate cancer, kidney cancer, Breast cancer, Lung cancer, bladder cancer, Pancreatic cancer, Colorectal cancer, Thyroide cancer, Qastric cancer	CXCR4-mediated signaling events, organism-specific biosystem Pathway Interaction Database ID: [cxcr4_pathway]
214247_s_at	DKK3				Lung cancer, prostate cancer, breast cancer, Head and neck cell carcinoma	
201137_s_at	HLA-DPB1				hepatitis C virus (HCV).	

## Discussion

### Biological interpretation

As observed from the biological details listed in Table 1, it is clear that top ten genes selected by the Pearson correlation coefficient and Cosine coefficient are the same. This is due to the high similarity between them according to Equations 1 and 3. Six of the top ten genes selected using these two methods have been previously implicated in different types of cancers according to their oncology term. Four genes (RGS1, LGALS3, IL7R and C7) have immune response process as one of their GO processes. Moreover, two genes (IL7R and LGALS3) are related to liver disease as they were manipulated in the literature of HCV and HCC. This derives the attention to the importance of carrying up further biological investigation on these discovered genes.

In Table 2, a different set of genes were retrieved using Mutual information. Four genes (DAB2, DCN, CELF2 and COL1A2) have appeared previously in cancers. Another gene (GPNMB) appeared to be involved with the liver inflammation. Two genes (DCN and C7) as shown in Table 3 are common genes in the list retrieved using Euclidean distance and Pearson's correlation coefficient methods. These two genes have been involved in colon and breast carcinoma. DAB2 which is a regulator of ovarian carcinoma and has been implicated in prostate and breast cancer is a common gene in the top ten genes selected using Euclidean distance and Mutual information.

The last method, Entropy using Empirical estimator, gave the lowest performance as shown in Table 4. Only one gene (DDX5) of the top ten genes has been previously implicated in prostate cancer.

For genes selected by the proposed hybrid technique, we can notice from Table 5 that there are 7 genes (2 genes for HCC and 5 genes in different types of cancer) are yielded. High portion of genes (LGALS3, CELF2, CYTIP, C7, DCN, CXCR4) retrieved by Pearson's correlation coefficient appeared in the top ten list retrieved by the hybrid technique. One gene (DCN) is common in the top ten list retrieved by Pearson's correlation coefficient, Mutual information, Euclidean distance and the proposed hybrid technique. This reveals the high importance of these genes as candidate biomarker for HCC.

### Examination of signal profiles

**Figure 3:**
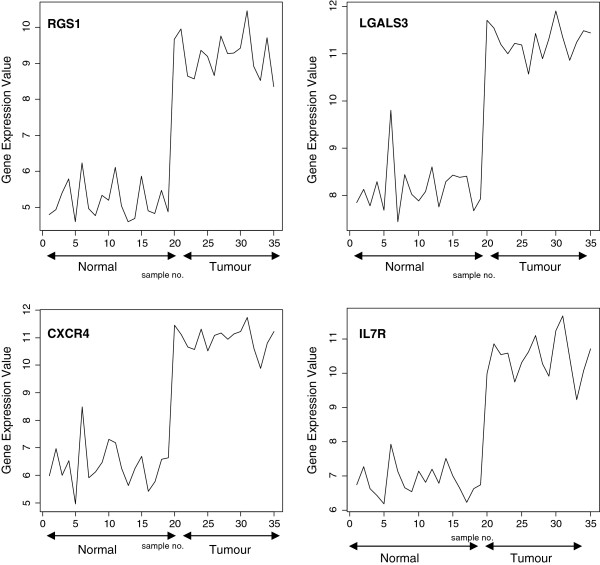
Profiles of four genes of top ten genes selected using Pearson's correlation coefficient and Cosine coefficient.

**Figure 4:**
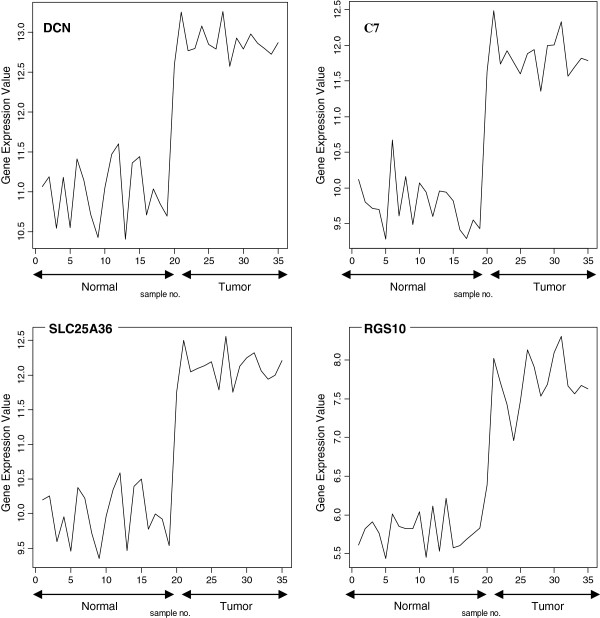
Profiles of four genes of top ten genes selected using Euclidean distance.

**Figure 5:**
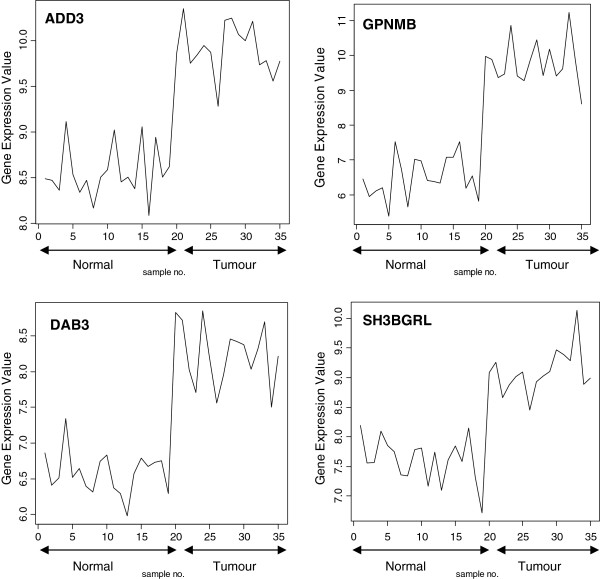
Profiles of four genes of top ten genes selected using Mutual information.

The signal profiles charted in Figures 3, 4 and 5 of genes selected using Pearson's correlation coefficient, Cosine coefficient; Euclidean distance and Mutual information show a high similarity with the signal profile of the ideal biomarker. In these figures, all signals have a step up shape. This derives us to consider these genes as up regulated biomarkers for HCC. The significance level of these signals according to their p-values in Table 6 shows the significance of the selected genes.

**Table 6:** The significance level of the first four genes in the top ten list

**Gene selection method**	**Gene symbol**	***t*-test**	**p-value**
**Pearson's Correlation Coefficient**	RGS1	−21.4863	<2.2e-16
	LGALS3	−21.6676	<2.2e-16
	CXCR4	−21.6883	<2.2e-16
	IL7R	−19.8349	<2.2e-16
**Euclidean Distance**	DCN	−18.8684	<2.2e-16
	C7	−19.5595	<2.2e-16
	SLC25A36	−19.9836	<2.2e-16
	RGS10	−14.6328	2.630e-12
**Mutual Information**	ADD3	−14.163	2.103e-15
	GPNMB	−15.6387	2.999e-16
	DAB3	−13.1599	5.279e-13
	SH3BGRL	−11.7037	4.181e-13
**Empirical Estimator**	HSPA7	0.1818	0.857
	EPHB3	4.9734	1.997e-05
	DDX5	−5.4255	6.336e-06
	Taf10	8.9728	3.129e-10
**Miller-Madow Entropy estimator**	HSPA7	0.1818	0.857
	EPHB3	4.9734	1.997e-05
	DDX5	−5.4255	6.336e-06
	Taf10	8.9728	3.129e-10
**Shrink Entropy estimator**	DDR1	−3.8611	0.0005685
	RFC2	4.8055	3.665e-05
	HSPA7	0.1818	0.857
	EPHB3	4.9734	1.997e-05
**Schurmann-Grassburger Entropy estimator**	HSPA7	0.1818	0.857
	EPHB3	4.9734	1.997e-05
	DDX5	−5.4255	6.336e-06
	Taf10	8.9728	3.129e-10

**Figure 6:**
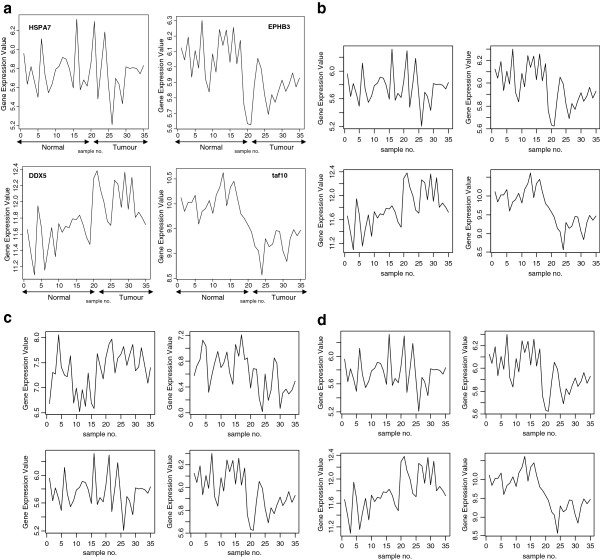
**(a) Profiles of four genes of top ten genes selected using Empirical entropy estimator.** (**b**) Profiles of four genes of top ten genes selected using Miller-Madow entropy estimator. (**c**) Profiles of four genes of top ten genes selected using Shrink entropy estimator. (**d**) Profiles of four genes of top ten genes selected using Schurmann-Grassburger Entropy estimator.

The signal profiles of genes selected using Entropy using Empirical estimator shown in Figure 6a have a random shape. Also as seen in Figure 6b, 6c and 6d, the other estimators of Entropy: Miller-Madow, Shrink, Schurmann-Grassberger gave a random shapes too for their selected genes. These signals have comparable p-values as in Table 6. These results reveal a low performance of Entropy. Without generalization, we conclude that the Entropy is an inadequate gene selection method in our application.

## Conclusions

The investigated methods of gene selection when used individually gave a comparable performance while Entropy method provided fairly acceptable result with respect to validating the results using the biological databases. Even more, by examining the signal profiles of genes retrieved using Entropy with different estimators, the signals are found to be visually non discriminating normal and tumour samples and this inspection was emphasised using *t*-test statistics. Nevertheless, the proposed hybrid approach surpassed all of the proposed methods when used solely. We conclude that the proposed technique is feasible when mining in a huge number of genes in high throughput microarrays. The proposed framework has detected a list of only 172 informative genes out of 22777 genes being studied. This list contains novel informative genes that are suggested as biomarkers for HCC and thus deserve further lab investigation as a future work.

## Competing interest

The authors declare that they have no competing interest.

## Authors’ contributions

NA collected the biological data, participated in data implementation and drafted the manuscript. NS and YK supervised the study, and participated in its design and coordination. All authors read and approved the final manuscript.
